# Genotypic and environmental effects on quality and nutritional attributes of Moroccan barley cultivars and elite breeding lines

**DOI:** 10.3389/fnut.2023.1204572

**Published:** 2023-10-12

**Authors:** Fadwa Elouadi, Ahmed Amri, Adil El-Baouchi, Zakaria Kehel, Abderrazek Jilal, Mohammed Ibriz

**Affiliations:** ^1^Plant Animal Productions and Agro-Industry Laboratory, Ibn Tofail University, Kenitra, Morocco; ^2^International Center for Agricultural Research in the Dry Areas (ICARDA), Rabat, Morocco; ^3^AgroBioSciences, Mohammed VI Polytechnic University, Ben Guerir, Morocco; ^4^National Institute for Agricultural Research, Regional Center of Rabat, Rabat Institutes, Rabat, Morocco

**Keywords:** barley, food, nutritional value, β-glucan, micronutrients, genetic × environment, stability analysis, GGE biplot

## Abstract

Although barley is mainly used for livestock feed and beverages, its use as human feed can enrich human diets with some health benefits. The development of new hulless varieties rich in β-glucans and micronutrients can enhance the use of barley as food, but little is known about the effects of the environment on these nutritional traits. In this study, we evaluated five Moroccan varieties and two elite breeding lines of barley at four locations in Morocco during the 2016–2017 and 2017–2018 cropping seasons. The results showed highly significant differences between genotypes for β-glucan, protein, iron, and selenium contents, as well as 1000 kernel weight, but not zinc content; significant to highly significant differences between environments for all traits except β-glucan content; and significant to highly significant interactions for all traits. The highest level of β-glucan content has reached 11.57% observed at the Sidi El Aydi site during the growing season 2017–2018 for the hulless variety Chifaa. This variety has shown the highest content of β-glucan (6.2–11.57%) over all environments except at Tassaout during the 2016–2017 seasons. The breeding line M9V5 has achieved significantly higher protein content at all the locations during the two growing seasons, ranging from 12.38 to 20.14%. Most hulless lines had significantly higher β-glucan and protein contents, but lower 1000 kernel weight. For micronutrients, the content ranges were 28.94 to 38.23 ppm for Fe, 28.78 to 36.49 ppm for Zn, and 0.14 to 0.18 ppm for Se, with the highest content for Fe and Zn shown by the breeding line M9V5 and Chifaa showing average contents of 33.39 ppm, 35.34 ppm, and 0.18 ppm for Fe, Zn, and Se, respectively. The GGE biplot confirmed the high and relatively stable content of β-glucan and acceptable micronutrient contents of the Chifaa variety and identified Marchouch as the most discriminant site to breed for biofortified barley varieties.

## 1. Introduction

Barley (*Hordeum vulgare* L) is considered one of the founding crops of old-world agriculture. Its cultivation began 10,000 years ago in the “Fertile Crescent” (1). It is one of the most adapted crops that can be grown under diverse climatic and edaphic conditions due to its ability to escape late drought and its salinity tolerance compared to other cereal crops (2).

Barley was a staple food crop for several thousand years, extending from Ethiopia and Egypt across the southern coast of the Mediterranean Sea to Morocco (3). Barley has played a major role in providing food security in Morocco throughout history; large grain storage has been maintained to ensure security by the ruling dynasties since the beginning of the second millennium BC (4).

The proven health benefits of barley have been the main driver for the renewed interest in the use of barley as food. Its health claims include the potential to reduce the risk of cardiovascular disease by lowering blood cholesterol (5). Barley has been preferred not only for its nutritional profile but also for its high concentrations of several nutraceutical compounds that have been shown to play an important role in functional foods (6). Barley is also a great source of many phytochemicals, including phenolics, phytates, and tocols, whose activity reduces oxidative degenerative reactions in the human body as evidenced by clinical studies (7).

The major components of barley grain are starch, dietary fiber, and protein, the contents of which are affected by genotypic and environmental factors as well as their interactions (8). It has been reported that among cereals, barley has a greater amount of soluble dietary fiber, particularly mixed linkage β-D-glucan, known for its health benefits (8). β-D-glucans are polysaccharides found principally in the cell walls of the aleurone layer and endosperm in both barley and oat kernels; they are more concentrated in the endosperm in barley, while in oats they are more concentrated in the aleurone layer (6). Changes in β-glucan levels have been attributed most of the time to environmental conditions, but the genotypic effect is also important (6). Increased levels of β-D-glucan have been correlated with dry and hot environmental growing conditions and the opposite when the environmental growing conditions are moist, such as rains during ripening (6). The variation in β-D-glucan is genetically associated with starch and with hulless cultivars known to have higher β-glucan content (9). A great diversity is reported in barley kernel colors at maturity, white, yellow, blue, purple, green, brown, and black pigmentation, which is usually due to the phenolic compound and anthocyanin content that give a different pigment in the pericarp and aleurone layer (10).

The use of barley in the starch industry generates proteins as a by-product that could be used in the food industry (11). Barley kernels are composed of 10–20% of proteins, mainly prolamin hordeins, which represent 87% of storage proteins in the endosperm layer, and glutenins, which represent 23% (12). Barley proteins are considered a good source of essential and non-essential amino acids (12). The protein content is under genetic control, but its expression is affected by the environment (13). The protein content is increased when nitrogen is supplied and under the effects of drought and heat stress (13).

In addition to its high-soluble fiber content, barley has other constituents with nutritional benefits, including vitamins (B1, B3,…) and micronutrients (calcium, iron, magnesium, phosphorus, potassium, zinc, and copper) (14). Indeed, zinc and iron deficiencies along with iodine, selenium, and vitamin A deficiencies are among the “big five” micronutrient deficiencies in humans throughout the world, responsible for over 65% of childhood deaths (15). Two billion people throughout the world are affected by micronutrient malnutrition also known as “hidden hunger,” which is common in resource-poor parts of the world where alternative food is rare to supplement the predominant cereal-based food (14). Furthermore, the cereal grown in mineral-deficient soils is very poor in both concentration and bioavailability of iron and zinc (16). Abraha et al. (17) reported in Ethiopian barley that the uptake of micronutrients and their translocation into grain are heritable traits. Mamo et al. (14) have reported a high heritability of Fe, Zn, and Se contents under greenhouse conditions; however, Joshi et al. (18) reported a low heritability under field conditions. For wheat, the major source of phenotypic variation for micronutrients is explained by the genotype ^*^ environment interaction (19).

To overcome micronutrient deficiencies, many strategies have been implemented, including supplementation, dietary diversification, fortification, and biofortification of crop plants (20). Compared to the other techniques, biofortification is considered more sustainable as it aims to increase genetically the bioavailable mineral content of food crops (21). Tools have been used to develop micronutrient-enriched cereals using genetic and genomic approaches (22).

Biofortification is either achieved through an agronomic approach, or genetic selection using conventional plant breeding methods, or biotechnology techniques (23). The agronomic approach is achieved by the application of foliar micronutrient fertilizers to cereal crops, but other strategies could be applied, such as crop rotation, correcting soil alkalinity, and the introduction of beneficial soil microorganisms (24–26). Barley biofortification through fertilization was applied to increase its bioavailable zinc and iron contents in the grains (27).

Genetic resources of cereal crops, including their wild relatives stored in gene banks, are a valuable source of alleles used in the improvement of crops, including their nutritional attributes (21). Screening of landraces and wild relative species is essential to identifying sources of micronutrients and other nutritional compounds. In this regard, the accessions of *Hordeum spontaneum* are a great source of high contents of β-glucans and micronutrients (28). Many genomic regions have been associated with richness in β-glucans and micronutrients, most of which are linked to wild alleles (29).

In crop improvement, the efficiency of selection is hindered by the magnitude of genotype–environment interactions. Breeders are using multi-environment testing to select varieties with good stability in the expression of the desired trait. There are several statistical methods used to characterize the stability and adaptation of varieties (30, 31), including the additive main effects and multiplicative interaction (AMMI) analysis, which is used for the stability analysis of zinc and iron in maize (32, 33). However, the genotype main effect plus genotype-by-environment interaction (GGE) biplot is increasingly used as it allows for better visualization of the performance and stability of genotypes, the discrimination ability and representativeness of environments, and the correlations between environments (34).

The aims of this study are: (i) to capture the effects of genotypes, environments, and their interactions on nutritional and quality attributes of some Moroccan barley varieties and elite lines, (ii) to identify barley varieties with higher nutritional value for human consumption, and (iii) to recommend testing environments for efficient breeding for quality and nutritional attributes in barley.

## 2. Materials and methods

### 2.1. Plant material and field trial

Five Moroccan barley varieties (Hulless and hulled, six-row and two-row), along with two breeding lines (green and dark brown aleurone), were obtained from the National Barley Breeding Program of the National Agricultural Research Institute of Morocco (INRA) (Table 1).

**Table 1:** Characteristics of barley varieties and breeding lines used.

**Variety**	**Row type**	**Grain type**	**Year of release**	**Origin**
Amalou	6	Hulled	1997	Morocco
Assiya	6	Hulless	2016	Morocco
Adrar	2	Hulled	1998	Morocco
Chifaa	6	Hulless	2016	Morocco
Rabat071	6	Hulled	1982	Morocco
M9V5	2	Hulless	Breeding line	ICARDA
BFH129	2	Hulless	Breeding line	ICARDA

These entries were grown in two replications in a randomized complete block design at each of the four Moroccan locations (Marchouch, Annoceur, Jamaat Shaim, and Tassaout) during the 2016–2017 cropping season and at Marchouch, Annoceur, Jamaat Shaim, and Sidi El Aydi during the 2017–2018 cropping season. Table 2 summarizes the geographic coordinates, altitude, and the agro-climatic zones of the locations. The trials were planted between mid-November and mid-December except at Tassaout, where planting was done on January 5, and was conducted based on the recommended agronomic packages, including the use of fertilizers and weeding using both herbicides and manually. The grains were harvested in the four central rows (5 m^2^) of each experimental unit, consisting of six rows of 5 m length and 0.25 m row spacing.

**Table 2:** Coordinates and eco-geographic zones of different testing locations.

**INRA experimental station**	**Coordinates**		**Agro-ecological zone**	
	**Latitude**	**Longitude**	**Zone**	**Altitude (m)**
Annoceur	33.667	−4.850	Mountain	1434
Jamaat Shaim	32.350	−8.850	Semi-arid	170
Marchouch	33.98	−6.49	Favorable	380
Sidi El Aydi	33.167	−7.400	Semi-arid	321
Tassaout	31.420	−6.467	Arid-Irrigated	460

The soil characteristics across the studied sites exhibit distinct patterns. Jamaat Shaim and Sidi El Aydi sites are characterized by clay soil texture, while Marchouch has heavy clay soil. Conversely, the Annoceur and Tassaout sites exhibited a medium texture with sandy clay loam soils. The presence of a calcium content risk is evident in all soils except at the Marchouch site. In terms of pH levels, all soils tend to be slightly basic to basic, except for the soil at Jamaat Shaim, where the pH remains neutral. Importantly, none of the soils are saline. The soils at Jamaat Shaim and Marchouch sites have medium organic matter content, while those at the other stations are poorer. The soils at the Marchouch and Sidi El Aydi sites exhibit high contents of available phosphorus, while Annoceur, Jamaat Shaim, and Tassaout sites display medium contents. Notably, all sites feature high available potassium content (more than 300 ppm), except for the soil at the Marchouch site (110 ppm).

**Figure 1:**
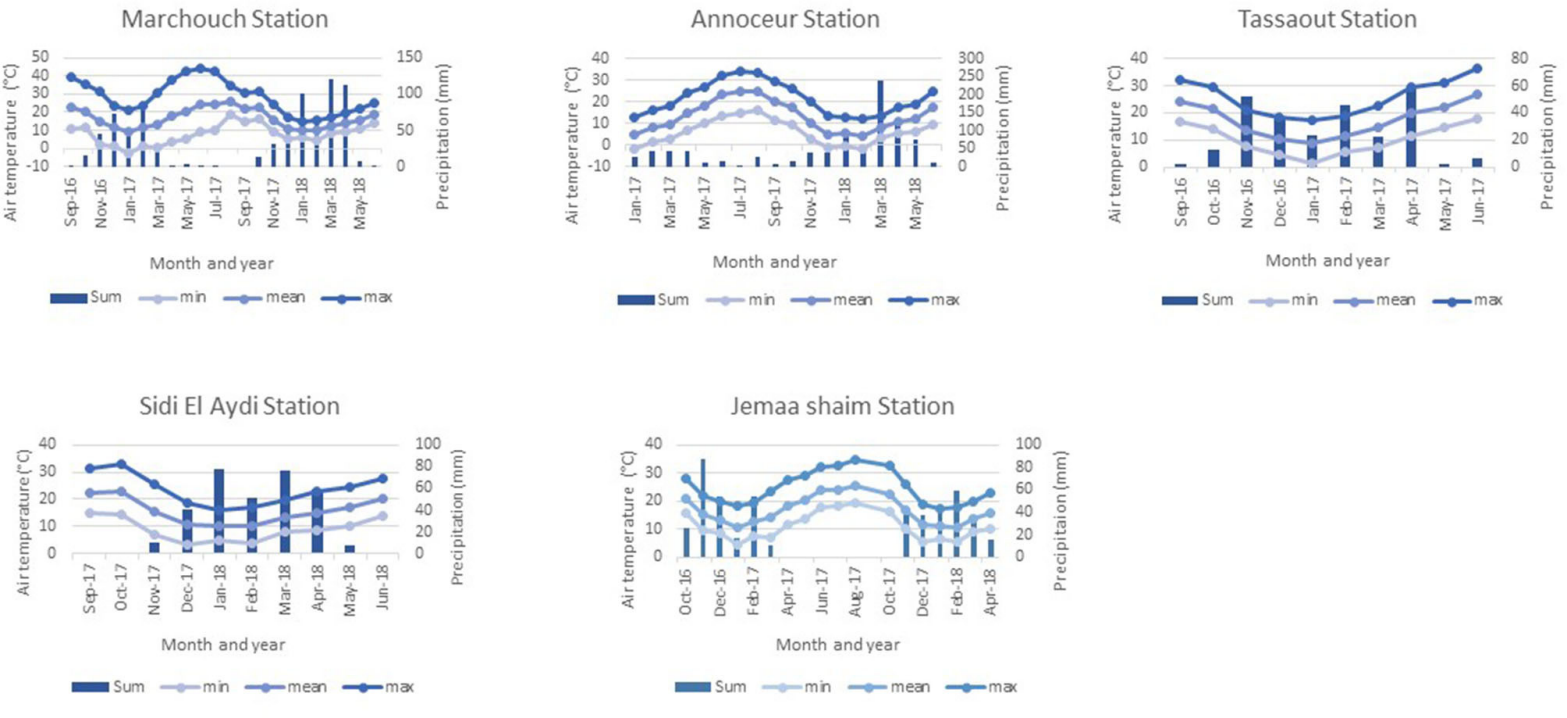
Monthly mean, maximum, minimum temperatures, and sums of precipitation of the testing locations during the two growing seasons 2016–2018.

Meteorological data for the experimental locations have been collected from the National Institute for Agriculture Research (Figure 1). Cumulative annual precipitation ranged from 286.6 mm in the first growing season to 497.4 mm in the second growing season for Marchouch station, from 204.8 mm (from January 2017 to August 2017) to 712 mm (from September 2017 to June 2018) for Annoceur station, and from 249 mm in the first growing season to 240 mm during the second growing season for Jemaa Shaim station. Higher temperatures were observed between May and September at the three locations. Cumulative precipitation in the other experimental locations was 262.8 mm and 325 mm, respectively, for Tassaout in 2016–2017 and Sidi El Aydi in 2017–2018. Only the trials at Tassaout received two supplemental irrigations of 20 mm each at sowing and at late tillering.

### 2.2. Grain sample preparation

Dried and cleaned grains were ground using a laboratory cyclone mill (Retsch Cyclone Mill Twister, Retsch, Haan, Germany) with a sieve of 1 mm. The grinding mill was cleaned before and after milling each sample to avoid cross-contamination. The moisture content of each sample was determined following the American Association of Cereal Chemists (AACC) approved method [44–40]-44–40.01 Moisture Modified Vacuum-Oven Method (35).

### 2.3. Beta glucan determination

The β-glucan content was determined according to a fluorometric method using a continuous flow analyzer (San ++ SKALAR, Breda, Netherlands). Prior to this determination, an acid extraction was performed using the procedure recommended by the European Brewery Convention (36). The extract was pumped and injected in the β-glucan module alongside the calcofluor reagent prepared previously and mixed, and then the mixture passed through the flow cell of the fluorimeter for the lecture. The signal was fed to a computer equipped with a specific software analysis system (FlowAccess 3.4.0, Skalar, Breda, Netherlands), and quantification was based on the calibration curve. The calibration curve is generated using known standards with known β-glucan concentrations. The software uses this curve to convert the measured signal to a corresponding β-glucan concentration in the sample.

### 2.4. Minerals' determination

The determination of microelement content was performed according to a standard protocol (37). The elemental determination was carried out with a simultaneous multi-element inductively coupled plasma-optical emission spectroscopy (ICP-OES) (ICAP-OES Duo, ICAP 7600 Series. Thermo Fisher Scientific, Waltham, USA), which detects ~30 elements simultaneously, including iron, zinc, and selenium.

### 2.5. Thousand kernel weight and protein determination

The thousand kernel weight was evaluated by weighing samples of 1000 kernels from the harvest of each plot, and the protein content was determined on the whole grain using near-infrared reflectance (NIR) (NIRS DS 2500, FOSS, Hillerod, Denmark) spectroscopy according to the AACC approved method [39–10] (35).

### 2.6. Statistical analysis

The analyses of variance for combined environments (location × year) and for each of the eight environments were conducted. The descriptive statistics, including the mean, LSD (5%), and CV (%), and tests of normal distribution were conducted for each trait in each environment. Data were also subject to GGE analysis, which uses principal component analysis to explain the maximum amount of variance in the original data by a few linearly transformed uncorrelated components. The resulting scatter plot based on PC1 and PC2 allows the visualization of mega-environments, the mean and stability of traits for each genotype and its comparison with the ideal genotype, the discriminating ability and representativeness of the environments, and the correlations among genotypes and environments (34). For each trait, similar genotypes are positioned closely in the biplot; genotypes far from the origin have large genotype plus interaction effects; and genotypes close to the origin have average performance in all environments. Closely associated environments suggest redundant locations, while large gaps indicate the need to extend the test environments to other locations. The plot ranking environments allow the identification of the most discriminant environment, which is nearest to the smallest circle representing the ideal environment. The same analysis can be conducted to compare the genotypes evaluated with the ideal genotype. The cosine of the angle between the vectors of genotypes or environments to the origin approximates the correlations between them; the acute angle shows a strong positive correlation, the obtuse angle shows a negative correlation, and the right angle indicates no correlation. Pearson's correlations were performed to assess relationships between traits. All statistical analyses were conducted using the GenStat software (19th Edition GenStat, VSNi, Hemel Hempstead, England) (38).

## 3. Results

### 3.1. Analysis of variance

The combined analysis of variance showed highly significant differences among entries for all measured traits except for zinc content (Table 3). Furthermore, the effect of the environment was highly significant for all traits except for β-glucan content. The interactions between entry × environment were highly significant for β-glucan, TKW, iron, zinc, and selenium and significant for protein content.

**Table 3:** Analysis of variance for quality attributes and TKW of barley varieties and breeding lines grown in eight environments.

**Source of variation**	**Df**	**β-glucan**	**Protein**	**TKW**	**Fe**	**Zn**	**Se**
Entry	6	265.92^***^ (55.52%)	150.73^***^ (20.09%)	2750.01^***^ (42.40%)	1046.96^***^ (11.23%)	662.674 (15.57%)	0.0299^***^ (11.04%)
Environment (Envt)	7	52.39 NS (10.94%)	504.51^***^ (67.23%)	1943.42^*^ (34%)	4389.40^***^ (47.10%)	2070.552 ^***^ (48.65%)	0.0382^***^ (14.12%)
Entry^*^ Envt	42	108.72^***^ (22.70%)	53.05 ^*^ (7.07%)	922.35 ^***^ (18.30)	3058.71^***^ (32.82%)	954.204 ^*^ (22.42%)	0.1663^***^ (61.42%)
Residual	48	32.15	32.26	532.39	713.19	463.281	0.0346
Total	111	478.962	750.37	6194.35	9318.26	4256.05	0.2708

TKW, Thousand kernel weight; Fe, iron; Zn, zinc; Se, selenium.

* Significant;

*** Highly significant.

Because the interactions between entry × environment were significant to highly significant, an analysis of variance was conducted for each trait in each environment, which allowed comparison among the entries and the derivation of descriptive statistics.

For β-glucan, the highest content of 11.58% was obtained for the variety Chifaa at the Sidi El Aydi station in 2017–2018. This variety has also given the highest contents in all environments except Tassaout and Jamaat Shaim in 2016–2017, where the variety Assiya has given similar contents (Table 4). At Annoceur, in 2016–2017, the β-glucan content varied from 2.78% for Adrar to 7.37% for Chifaa, with 5.94% for the breeding line BFH129; during the 2017–2018 season, Adrar showed 6.02% of β-glucan, while Chifaa showed 9.04%. At Marchouch, the ranges were 1.95 to 8.78% in 2016–2017 and from 3.94 to 10.84% in 2017–2018, respectively, for the varieties Amalou and Chifaa. At Jamaat Shaim, the same ranking of these two varieties was observed during the two consecutive seasons, with Amalou having the lowest content and Chifaa the highest. At Tassaout, during 2016–2017, Assiya and Chifaa showed the highest content, while the lowest values were given by the varieties Amalou and Adrar.

**Table 4:** β-glucan content mean, CV, and LSD for barley entries in different environments.

β**-glucan (%)**									
**Entry**	**2016–2017**				**2017–2018**				**Mean**
	**Annoceur**	**Marchouch**	**Jamaat Shaim**	**Tassaout**	**Annoceur**	**Marchouch**	**Jamaat Shaim**	**Sidi El Aydi**	
Adrar	2.78	3.43	3.50	3.38	6.02	6.44	4.84	5.58	4.49
Amalou	4.61	1.95	1.88	2.54	3.72	3.94	2.89	4.72	3.28
Chifaa	7.35	8.78	6.20	6.69	9.04	10.84	7.56	11.58	8.50
Assiya	4.25	3.62	6.17	6.81	5.45	6.00	4.40	3.92	5.08
Rabat 071	3.23	2.78	3.22	5.88	3.60	5.16	4.91	5.13	4.24
BFH129	5.94	6.63	3.92	4.97	6.38	5.42	4.47	5.49	5.40
M9V5	3.01	5.27	3.36	3.74	4.47	5.70	5.26	4.14	4.37
Mean	4.45	4.64	4.04	4.86	5.53	6.21	4.90	5.79	
LSD (5%)	3.38	1.17	0.73	1.86	1.56	2.71	3.07	22.5	
CV (%)	21.1	10.7	7.6	17	12	18.5	26.5	16.4	

LSD, least significant difference; CV, coefficient of variation.

The protein content varied from 9.28% for the landrace Rabat 071 at Annoceur in 2017–2018 to 20.14 for the breeding line M9V5 at Marchouch in 2016–2017 (Table 5). The highest protein concentrations were registered at Marchouch, Jamaat Shaim, and Tassaout in 2016–2017 and at Marchouch and Sidi El Aydi in 2017–2018. Annoceur in 2017–2018 showed the lowest protein percentage. The breeding line M9V5 had the highest value in each of the eight environments and ranged from 12.38% to 20.14%, followed by the variety Assiya (10.10 to 18.07%) and the breeding line BFH129 (11.15 to 16.49%). In most environments, the varieties Adrar and Amalou had low protein content, while Rabat 071 and Chifaa, Assiya, and the breeding line BFH129 had intermediate protein content.

**Table 5:** Protein content mean, CV, and LSD for barley entries in different environments.

**Protein**									
**Entry**	**2016–2017**				**2017–2018**				**Mean**
	**Annoceur**	**Marchouch**	**Jamaat Shaim**	**Tassaout**	**Annoceur**	**Marchouch**	**Jamaat Shaim**	**Sidi El Aydi**	
Adrar	12.51	17.02	14.94	15.2	10.15	14.36	13.83	15.57	14.2
Amalou	13.26	17.2	14.59	14.55	9.93	14.18	14.56	15.87	14.27
Chifaa	15.73	17.34	16.64	17.2	10.41	14.35	15.62	16.6	15.49
Assiya	14.49	18.77	18.07	17.82	10.1	14.8	16.09	17.15	15.91
Rabat 071	14.77	17.64	17.86	16.82	9.28	15.15	16.5	15.49	15.44
BFH129	14.21	17.38	16.49	14.2	11.15	16	14.95	16.59	15.12
M9V5	18.17	20.14	19.98	19.86	12.38	16.59	17.62	18.68	17.93
Mean	14.73	17.92	16.94	16.52	10.49	15.06	15.6	16.56	
LSD (5%)	1.35	0.94	1.73	3.62	1.33	0.82	2.01	2.88	
CV (%)	3.9	2.2	4.3	9.3	5.4	2.3	5.5	7.4	

LSD, Least significant difference; CV, coefficient of variation.

For the thousand kernel weight, the highest values were recorded in the varieties Rabat 071 (41.13 to 59.30 g) and Adrar (42.46 to 54.31 g), while the lowest values were given by the breeding line M9V5 (30.15 to 39.88 g) and the variety Assiya (27.87–41.02 g) over the eight environments (Table 6). The remaining entries have a medium grain weight. The highest TKW was recorded at Annoceur in 2017–2018, followed by Tassaout in 2016–2017 and Marchouch in 2017–2018, while the lowest values were recorded at Marchouch in 2016–2017 and Jamaat Shaim and Sidi El Aydi in 2017–2018.

**Table 6:** TKW mean, CV, and LSD for barley entries in different environments.

**TKW (g)**									
**Entry**	**2016–2017**				**2017–2018**				**Mean**
	**Annoceur**	**Marchouch**	**Jamaat Shaim**	**Tassaout**	**Annoceur**	**Marchouch**	**Jamaat Shaim**	**Sidi El Aydi**	
Adrar	43.66	43.74	43.65	48.77	54.31	46.89	42.56	52.74	47.04
Amalou	44.66	30.59	35.99	41.69	45.92	39.13	30.44	29.39	37.23
Chifaa	33.57	32.23	33.08	36.15	42.05	41.93	33.76	35.43	36.03
Assiya	34.23	27.87	34.42	41.02	35.53	37.05	33.89	34.52	34.81
Rabat 071	47.61	44.44	48.79	50.09	59.3	53.13	38.15	41.13	47.83
BFH129	45.81	39.17	43.21	47.36	47.73	40.96	37.31	29.77	41.42
M9V5	32.96	30.3	30.15	35.57	39.88	37.34	31.97	30.92	33.64
Mean	40.36	35.48	38.47	42.95	46.39	42.35	35.44	36.27	
LSD (5%)	7.35	2.34	2.24	5.94	2.95		9.34	12.98	
CV (%)	7.7	2.8	2.5	5.9	2.7		11.1	19.6	

LSD, Least significant difference; CV, coefficient of variation; TKW, thousand kernel weight.

Regarding the microelements, large variations were observed between entries and between environments. For iron content, it ranged from 17.49 ppm at Sidi El Aydi in 2017–2018 to 51.41 ppm at Marchouch in 2016–2017 for the same variety, Amalou (Table 7). It appeared that the iron contents were higher during the 2016–2017 season than in the 2017–2018 season. Among the entries, the varieties Adrar and Assiya had the lowest Fe content in most environments, while the breeding line M9V5 had higher values in the three environments in the 2017–2018 season. The breeding line BFH129, the landrace Rabat 071, and the varieties Amalou and Chifaa showed large variations with high to medium contents in most environments. For zinc content, the entries did not show significant differences when averaged over all environments. However, their content differed in some environments (Table 7). At the Marchouch station, the ranges were 29.15 ppm for Adrar to 42.96 ppm for Rabat 071 in the 2016–2017 season and from 25.08 ppm for Amalou to 34.28 ppm for Rabat 071 in 2017–2018. At Jamaat Shaim station, only the Adrar variety showed the lowest content of 21.86 ppm compared to the breeding line BHF129 (30.10 ppm) in 2016–2017, while no significant differences were declared among entries in 2017–2018. At Annoceur station, no significant differences were found among entries in 2016–2017, while in 2017–2018, the variety Adrar has given the lowest content of 23.18 ppm, while the rest of the entries have shown contents ranging between 38.21 and 42.82 ppm. For Sidi El Aydi in 2017–2018, no significant differences were found among entries. At Tassaout in 2016–2017, higher Zn contents were shown by the breeding line M9V5 (46.53 ppm) and the variety Chifaa (43.15 ppm) when compared with the variety Amalou (33.24 ppm). For selenium content, no significant differences were found between entries at Annoceur in 2016–2017, while the variety Amalou (0.09 ppm) showed significantly less than Chifaa (0.21 ppm) in 2017–2018 at the same station (Table 7). At the Marchouch site, the varieties Assiya, Chifaa, and Amalou outperformed the varieties Rabat 071 and Adrar, while the two breeding lines were intermediate in 2016–2017. In 2017–2018, the varieties Rabat 071 and Assiya showed by far the lowest values of 0.05 and 0.07 ppm, respectively, followed by the breeding line M9V5 (0.13 ppm) and Adrar (0.18 ppm), Chifaa (0.20 ppm), and Amalou (0.21 ppm), while the highest content was shown by the breeding line BFH129. At Jamaat Shaim, no significant differences were declared among entries in 2016–2017; however, large differences were found in 2017–2018, with a range of 0.07 ppm for the variety Assiya to the highest content given by the breeding line BFH129 (0.23 ppm), followed by the varieties Adrar (0.21 ppm) and Chifaa (0.20 ppm). At Tassaout in 2016–2017, the lowest content was registered for the variety Adrar and the highest by Rabat 071, while the remaining entries ranged from 0.15 to 0.17 ppm. At the Sidi El Aydi site in 2017–2018, the lowest content was shown by the breeding line M9V5 (0.06 ppm), followed by BFH129 (0.13 ppm), while the three varieties Adrar, Amalou, and Rabat 071 had a content of 0.21 ppm, and the hulless varieties Assiya and Chifaa had 016 ppm.

**Table 7:** Iron, zinc, and selenium contents mean, CV, and LSD for barley entries in different environments.

**Fe (ppm)**									
**Entry**	**2016–2017**				**2017–2018**				**Mean**
	**Annoceur**	**Marchouch**	**Jamaat Shaim**	**Tassaout**	**Annoceur**	**Marchouch**	**Jamaat Shaim**	**Sidi El Aydi**	
Adrar	36.67	40.33	37.87	24.37	23.07	22.61	26	21.55	29.06
Amalou	35.7	51.41	44.52	43.77	26.53	23.22	25.04	17.49	33.46
Chifaa	44.33	39.14	33.32	38.6	32.84	21.88	22.49	36.1	33.59
Assiya	43.31	18.95	31.1	29.16	26.03	33.75	24.87	24.37	28.94
Rabat 071	43.05	41.72	33.97	48.99	27.48	26.47	32.82	27.23	35.22
BFH129	45.91	38.64	43.98	41.21	22.69	22.14	25.68	31.16	33.92
M9V5	43.44	40.52	45.51	41.59	38.79	26.35	31.75	37.94	38.23
Mean	41.77	38.67	38.61	38.24	28.2	25.2	26.95	27.97	
LSD (5%)	11.61	6.68	10.43	13.03	4.5	8.78	8.91	4.77	
CV (%)	11.8	7.3	11.4	14.4	6.7	14.7	14	7.2	
**Zn (ppm)**									
**Entry**	**2016–2017**				**2017–2018**				**Mean**
	**Annoceur**	**Marchouch**	**Jamaat Shaim**	**Tassaout**	**Annoceur**	**Marchouch**	**Jamaat Shaim**	**Sidi El Aydi**	
Adrar	29.45	29.15	21.86	38.44	23.18	26.27	30.62	31.27	28.78
Amalou	29.18	34.14	26.68	33.24	42.31	25.08	29.82	37.87	32.29
Chifaa	33.72	37.23	25.45	43.15	40.51	28.32	32.61	41.76	35.34
Assiya	31.38	38.85	26.65	41.6	42.82	32.61	32.19	36.96	35.38
Rabat 071	30.14	42.96	26.09	37.08	41.47	34.28	33.36	30.8	34.52
BFH129	29.35	35.15	30.1	39.08	41.84	34.19	35.96	34.69	35.05
M9V5	34.02	41.37	29.15	46.53	38.21	32.13	32.24	38.25	36.49
Mean	31.03	36.98	26.57	39.87	38.62	30.41	32.4	35.94	
LSD (5%)	5.98	5.49	6.99	6.55	6.3	7.81	11.5	7.97	
CV (%)	8.1	6.3	11.1	7	6.9	10.9	15	9.4	
**Se (ppm)**									
**Entry**	**2016–2017**				**2017–2018**				**Mean**
	**Annoceur**	**Marchouch**	**Jamaat Shaim**	**Tassaout**	**Annoceur**	**Marchouch**	**Jamaat Shaim**	**Sidi El Aydi**	
Adrar	0.17	0.17	0.22	0.13	0.12	0.18	0.2	0.21	0.18
Amalou	0.17	0.24	0.16	0.14	0.09	0.21	0.13	0.21	0.17
Chifaa	0.14	0.24	0.17	0.14	0.21	0.2	0.21	0.16	0.18
Assiya	0.17	0.25	0.14	0.16	0.12	0.06	0.07	0.16	0.14
Rabat 071	0.14	0.17	0.14	0.22	0.14	0.07	0.16	0.21	0.16
BFH129	0.16	0.22	0.17	0.17	0.12	0.25	0.23	0.13	0.18
M9V5	0.16	0.18	0.14	0.15	0.18	0.13	0.13	0.06	0.14
Mean	0.16	0.21	0.16	0.16	0.14	0.16	0.16	0.16	
LSD (5%)	0.03	0.06	0.08	0.05	0.09	0.04	0.04	0.05	
CV (%)	7.3	12.3	22.1	14	26.6	11.5	11.1	13.5	

LSD, least significant difference; CV, coefficient of variation; Fe, iron; Zn, zinc; Se, selenium.

### 3.2. Pearson's correlations

Pearson's correlations between traits were only highly significant between TKW and protein content (-0.544) and significant between iron and β-glucan contents with a negative correlation of (−0.192) and between iron and protein with a positive correlation of (0.278) (Table 8). The correlations among the micronutrients were not significant.

**Table 8:** Correlation between quality and nutritional traits measured for seven barley entries over eight environments in Morocco.

	**β-glucan**	**Protein**	**TKW**	**Fe**	**Zn**	**Se**
β-glucan	1					
Protein	−0.053	1				
TKW	−0.024	−0.544^**^	1			
Fe	−0.192^*^	0.278^*^	−0.043	1		
Zn	0.109	0.028	−0.053	0.020	1	
Se	0.073	0.136	−0.151	−0.003	−0.071	1

** The correlation is significant at the 0.01 level;

* the correlation is significant at the 0.05 level. TKW, thousand kernel weight; Fe, iron; Zn, zinc; Se, selenium.

### 3.3. GGE biplot analysis

The GGE biplot analysis was conducted only for β-glucan and the micronutrient contents, and four different plots were selected for the purpose of this study: (i) the scatter plot of the “which-wins-where” pattern, which allows to identify different sectors by joining the winner genotype in each sector to identify different mega-environments and the best genotypes in each mega-environment; (ii) the ranking plot, which allows to evaluate the genotype performance and stability through the projection of the genotype performance on average environment coordinate (AEC) abscissa. The shorter the projection from the AEC is, the more stable the genotype is; (iii) the comparison plot, which allows to identify the environment with the most discriminating power among test environments; and (iv) the joint biplot, which allows to compare the variety Chifaa (recommended for human food) with the landrace Rabat 071, the widely grown landrace in Morocco.

**Figure 2:**
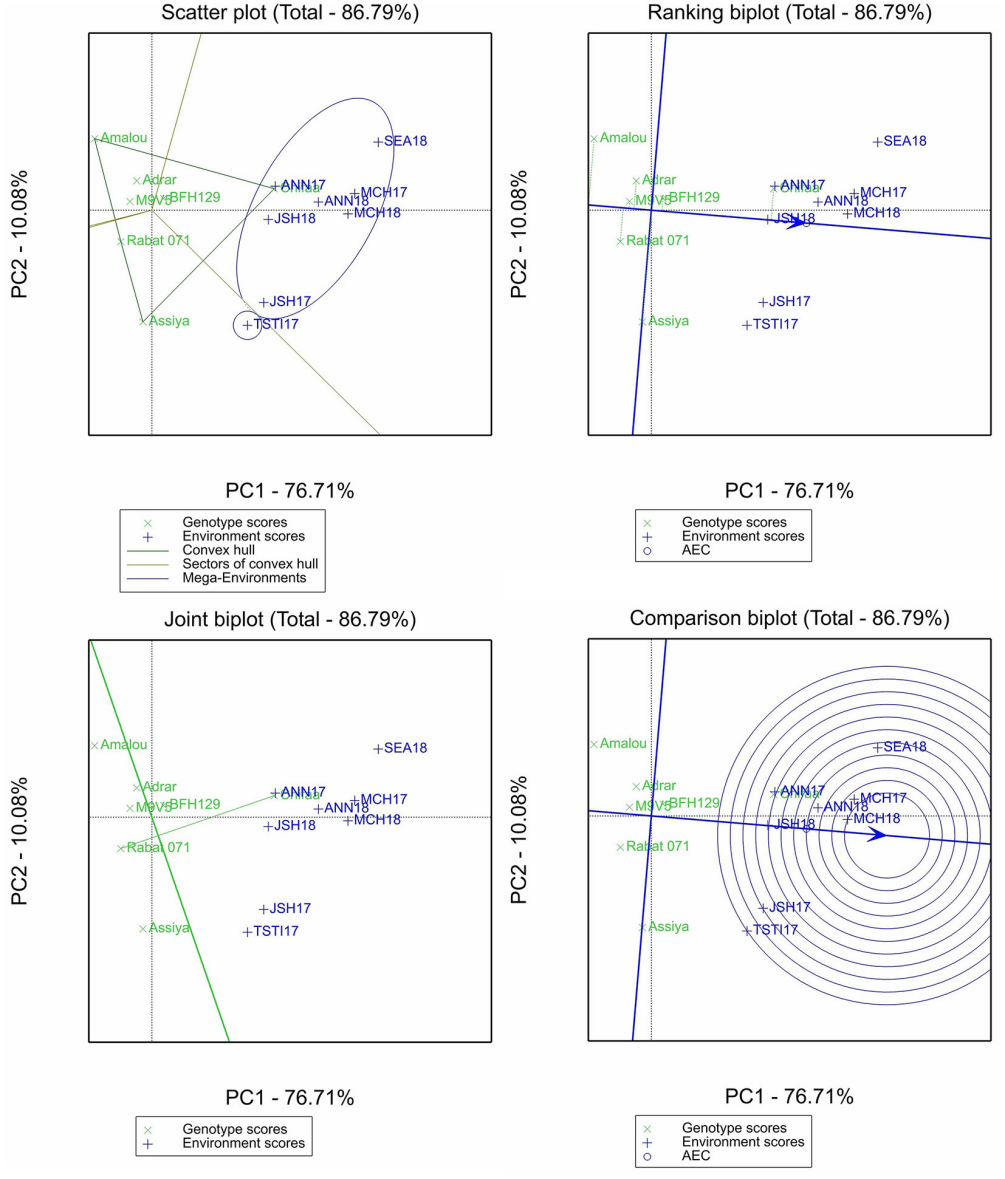
Scatter plot, ranking biplot, joint biplot, and comparison biplot for β-glucan of barley entries evaluated in eight environments (ANN, Annoceur; JSH, Jamaat Shaim; TST, Tassaout; MCH, Marchouch; and SEA, Sidi El Aydi).

The two principal components of the biplot analysis for β-glucan accounted for 87.79% of the total variation (Figure 2). The scatter plot identified two mega-environments, with all of Annoceur, Marchouch, Jamaat Shaim, and Sidi El Aydi in one and only Tassaout 2016–2017 environments in the other. The ranking biplot showed that the variety Chifaa has the highest β-glucan content in all environments with relatively good stability. The breeding line BFH129 had a higher content than the mean and a stable performance over the environments, while all the rest of the entries performed less than the average, with the varieties Assiya and Amalou being the least stable. The comparison plot showed that Marchouch's location was the most discriminant site, as both related environments were within the inner circle, representing the ideal environment. The joint biplot shows that Chifaa outperformed Rabat 071.

**Figure 3:**
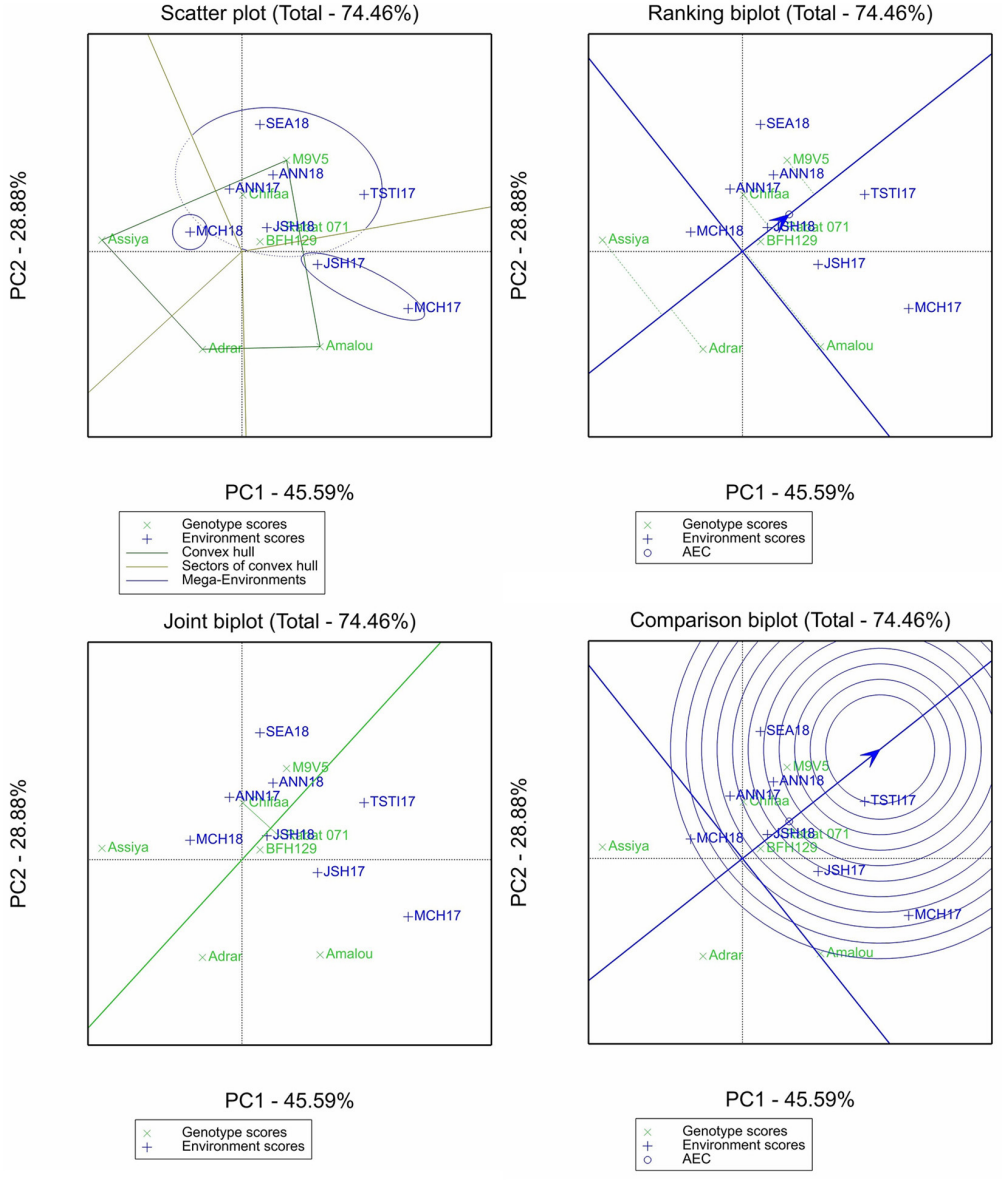
Scatter plot, ranking biplot, joint biplot, and comparison biplot for iron of barley entries evaluated in eight environments (ANN, Annoceur; JSH, Jamaat Shaim; TST, Tassaout; MCH, Marchouch; and SEA, Sidi El Aydi).

For iron content, the two first principal components explained 74.46% of the variation (Figure 3). The scatter plot identified three mega-environments with a vertex of four sectors, with Marchouch 2016–2017 and Jamaat Shaim 2016–2017 forming the first mega-environments, Marchouch 2017–2018 the second mega-environment, and the remaining environments forming the third mega-environment. The ranking plot showed that the varieties Adrar and Assiya performed less than the average genotype and were unstable, the variety Amalou had average content but was the most unstable, and the remaining entries performed more than the average genotype with more stable performances. The comparison biplot identified only Tassaout 2016–2017 as the most discriminant environment, while both Marchouch environments were the least discriminant for this trait. The variety Chifaa has a slightly higher content of iron than Rabat 071, which appeared more stable.

**Figure 4:**
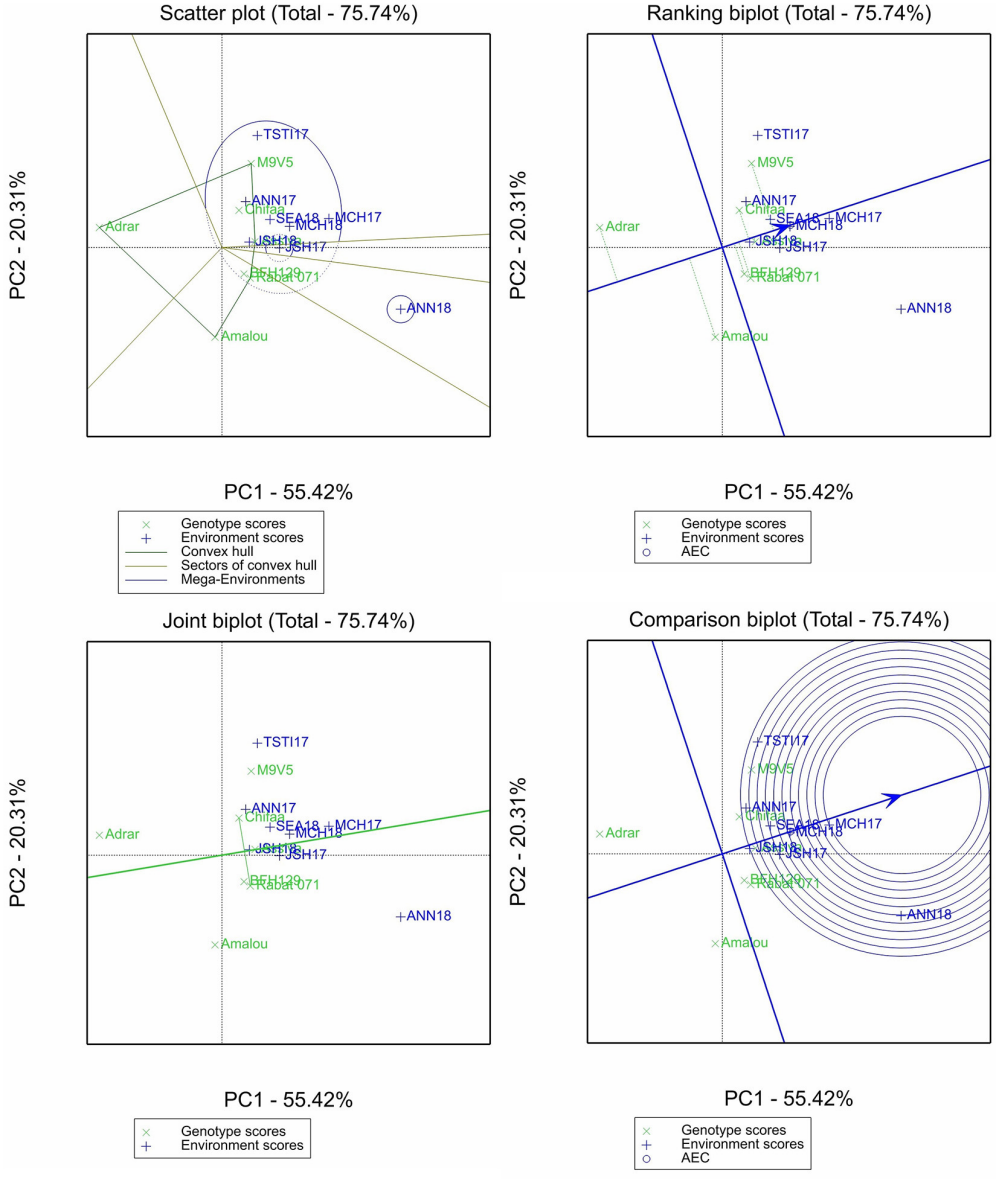
Scatter plot, ranking biplot, joint biplot, and comparison biplot for zinc of barley entries evaluated in eight environments (ANN, Annoceur; JSH, Jamaat Shaim; TST, Tassaout; MCH, Marchouch; and SEA, Sidi El Aydi).

For zinc content, 75.74% of the variation was accounted for by the two first principal components (Figure 4). Two mega-environments were identified, one having only Annoceur 2017–2018 and the other having the rest of the environments. Among the entries, the varieties Adrar and Amalou had the lowest zinc content but were the most unstable, while the remaining entries had above-average genotypes, with only the breeding line M9V5 being the most unstable. Marchouch in 2016–2017 allowed us to discriminate better among the entries for this trait. Chifaa slightly outperformed the landrace Rabat 071.

**Figure 5:**
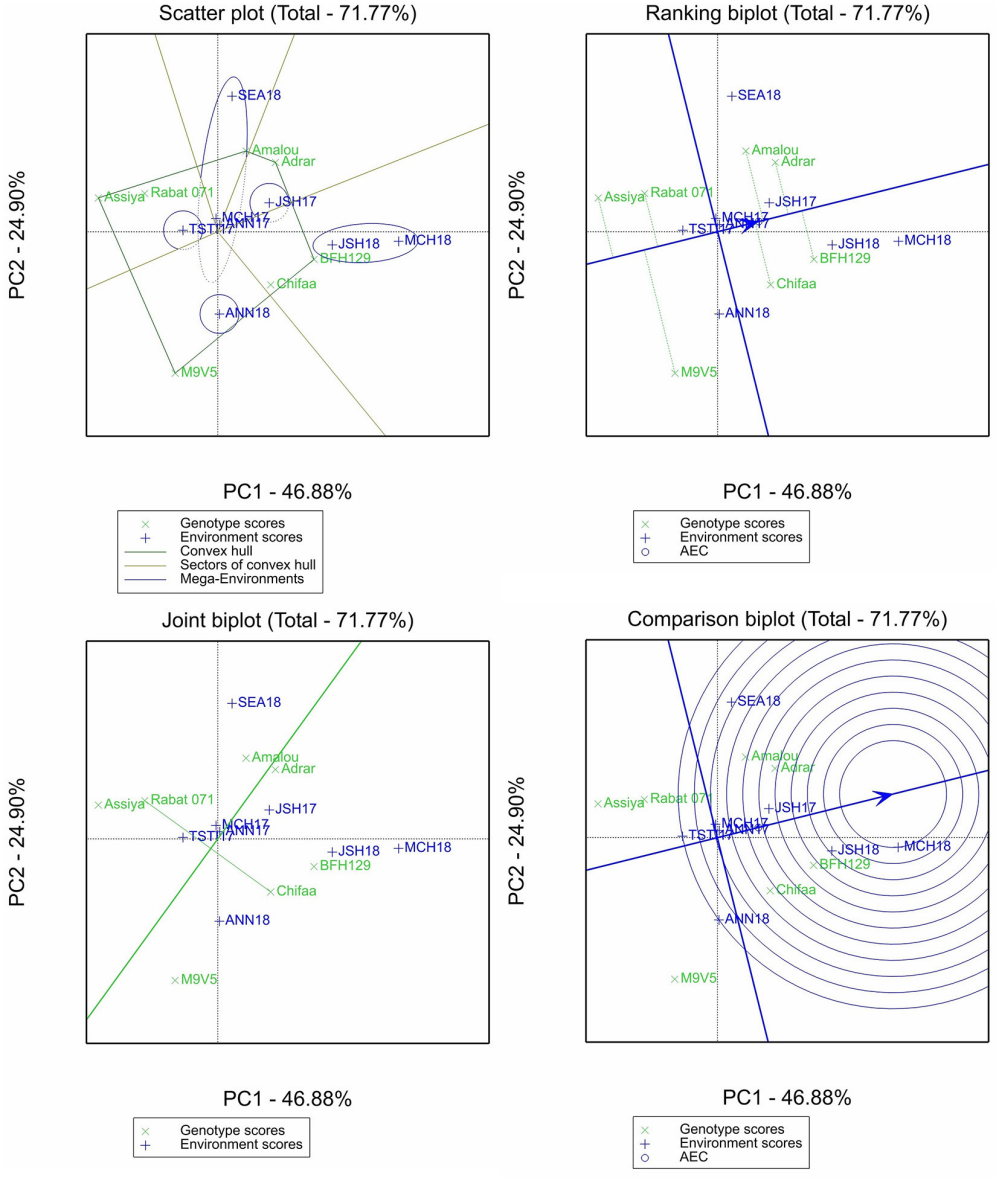
Scatter plot, ranking biplot, joint biplot, and comparison biplot for selenium of barley entries evaluated in eight environments (ANN, Annoceur; JSH, Jamaat Shaim; TST, Tassaout; MCH, Marchouch; and SEA, Sidi El Aydi).

For selenium content, the two first principal components explained 71.77% of the variation (Figure 5). The scatter plot allowed us to identify five mega-environments; one for each of the environments Annoceur 2017–2018, Jamaat Shaim 2016–2017, and Tassaout 2016–2017; one mega-environment including Jamaat Shaim and Marchouch in 2017–2018; and fifth mega-environment including the rest of the environments as shown by the scatter biplot. The ranking biplot classified the entries M9V5, Assiya, and Rabat 071 as having below-average content of selenium, with M9V5 being the most unstable entry overall. The remaining entries had above-average content and showed less stability of performance; Chifaa had higher content than Rabat 071.

## 4. Discussion

Micronutrient malnutrition, referred to as “hidden hunger,” affects over two billion people in the world, including those living under harsh conditions. Iron and zinc deficiencies are among the most common and are posing serious health challenges in developing countries, mainly for resource-poor farming communities. Morocco is known for its highest per capita consumption of barley as food in the world, mainly by the rural communities in the mountainous and arid areas. Due to the predominant cereal-based diets and the limitations of diversifying the diets, biofortification through breeding micronutrient-rich varieties has become a research priority in many crop breeding programs. Several studies have promoted genetic biofortification as a more cost-effective and sustainable approach compared to agronomic biofortification, food diversification, or supplements (39, 40). The HarvestPlus and Biofortification Challenge research programs in collaboration with CGIAR (The Consultative Group on International Agricultural Research) centers and other national partners were able to develop zinc-enriched varieties of maize, rice, and wheat; iron-enriched varieties of bean and pearl millet; and vitamin A-enriched varieties of cassava, maize, and sweet potato (https://www.harvestplus.org/home/crops). ICARDA and many countries, including Morocco, are working on the development of barley with high β-glucan, which allowed the release by INRA-Morocco of two hulless barley varieties, Assiya and Chifaa, with the latter having high β-glucan.

The results of the present study on five released barley varieties in Morocco and two breeding lines showed large genotypic differences in protein content, thousand kernel weight, β-glucan, and Fe and Se micronutrient contents, significant effects of environments except for β-glucan, and significant genotype × environment interactions for all traits, indicating the potential for selecting nutrient-enriched germplasm and the need for multi-environment testing for selecting more stable genotypes. Many authors have reported similar findings, highlighting the greater significance of genotypic effects over environmental effects on protein content (9, 41–43). A non-significant effect of environment is reported for Ethiopian barley for β-glucan and protein contents (17). A negative correlation between β-glucan content and accumulated temperature to 30°C and total precipitation during grain filling (13), as well as between β-glucan and protein contents in drought-stressed environments, was reported (17). Zhang et al. (13) reported that the variations in β-glucan and protein contents are mainly due to environmental factors. The Moroccan variety Chifaa has the highest levels of β-glucan in all environments, with a percentage higher than what is reported in other studies (44).

Several studies have shown that the protein content is mainly under genetic control, with a slicing effect of the environment (13) and a high genotype × environment interaction (45). In our study, the interactions between genotype × environment were significant for all traits, including protein content. Andersson and Börjesdotter (46) have reported that the protein content has significant correlations with rainfall and degree days.

In this study, among micronutrients, Zn appears to be influenced more by the environment, while Se showed a high influence of the environment and a high genotype × environment interaction. Significant genotypic and environmental effects and their interactions were also reported for micronutrient contents in wheat (47). In other studies, the variation of Fe and Zn contents in barley genotypes depended on the variation of environmental conditions (48), while they were under genetic control for Ethiopian barley (17). Xu et al. (32) reported that the concentrations of Fe and Zn in grains of maize under optimum conditions were higher compared to under low-N conditions. Among the micronutrients, Se was the least studied in barley despite its health benefits on thyroid function, and our study showed a high contribution of genotype × environment interactions to its variation, making the selection of this trait more difficult.

Thousand kernel weight is an important yield component and quality attribute of barley that is most appreciated by farmers. This study showed the predominance of genetic effects and high genotype × environment interactions. Similar conclusions were reported by Boudiar et al. (49) and Bocianowski et al. (50); however, Kumar et al. (51) did not report significant genotype × environment interactions for barley grown in India. Among all the traits evaluated, our results showed the highest negative correlation between TKW and protein content, while Fe showed the highest positive correlation with β-glucan and a negative correlation with protein content, but no significant correlations were found between the micronutrient contents. Other studies have reported significant correlations between Zn and Fe in Syrian barley (52), in wheat (53), and with protein content in wheat (54). β-glucan was reported to be correlated with TKW in oats (46). Based on these results, selection should be conducted independently for the micronutrients and for β-glucan, and the negative correlation between TKW and protein content should be considered during the selection. The identification of molecular markers could facilitate the selection for quality and nutritional traits if associated QTLs and markers are identified, as reported in barley for protein and β-glucan contents (55, 56).

Morocco lies within the Mediterranean region, known for high climate variability within and between the seasons. Therefore, a comprehensive evaluation of GE interactions requires more advanced statistical methods than the standard ANOVA. Since the genotype × environment interactions were significant to highly significant for all traits measured, selection and identification of lines with higher β-glucan and higher micronutrient contents will be facilitated by the identification of locations with the most discriminating power for plant selection during segregating populations and by the evaluation of the performance stability of the derived fixed lines using multi-environment testing. GGE biplot was used since it offers many features not shown in other stability analysis approaches, GGE biplot provides many visual interpretations, including crossover G × E interaction, comparing tested genotypes with ideal genotypes, and identifying environments with the most discriminating ability (57). In the GGE biplot, the first component is highly correlated with genotype performance, while the second component allows for assessing the variation of the trait due to genotype × environment. For β-glucan, most of the environments could be used to identify genotypes with high content, but the Marchouch station appears more appropriate for efficient selection for this trait in early and later generations. Additional locations will be needed to fit the two additional sectors identified through the scatter plot. Among the tested genotypes, the hulless barley variety Chifaa has the highest β-glucan content and can express its high β-glucan when grown in all environments. For iron content, except for Tassaout in 2016–2017, none of the environments has high discriminating power, and Marchouch station has the least discriminating ability. Among the genotypes tested, the varieties Assiya and Adrar showed the lowest mean content and highest instability and therefore cannot be used as biofortified varieties for this trait. However, the variety Chifaa and the hulless breeding line M9V5 have above-average iron content. For zinc content, all environments were grouped in one sector except for Annoceur 2017–2018, and none of the environments covered the remaining two sectors. Based on the ranking biplot, only the varieties Adrar and Amalou have low zinc content, while Chifaa and the breeding line M9V5 have above-average content but less stable content. Among the locations, Marchouch appears to be the most appropriate for the selection and evaluation of this trait. For selenium, all the genotypes tested showed high instability, but selection for high content could be done at Marchouch station.

Based on the results of the analysis of variance and stability analysis using the GGE biplot, the hulless barley varieties Chifaa and Assiya can be recommended for human consumption, with the former one having the advantage of being biofortified for β-glucan with acceptable levels of Fe, Zn, and Se contents. The two hulless breeding lines BFH129 and M9V5 can also be promoted as varieties for human consumption or used as sources of micronutrients to be crossed with Chifaa for the development of varieties combining high β-glucan and high content of micronutrients. For micronutrients, a special search for parental germplasm with high contents is needed to develop biofortified varieties. For the remaining varieties, the landrace Rabat 071 and the newly released varieties Amalou and Adrar can be considered for multiple purposes, but their use as human food will require a dehulling process which could reduce the micronutrients. Among the experimental stations used, Marchouch appears to be the most appropriate for selecting β-glucan glucans and for Se and Zn but not for Fe. As a breeding implication, this station can be used to select within segregating populations, but multilocational trials are needed for most advanced lines to select the ones combining high-quality and more stable varieties for quality and nutritional traits. For commercial production of biofortified varieties, it will be good to consider environments that allow the best expression of these nutritional traits. More research is needed on the stability and bioavailability of these nutrients during processing and consumption.

## 5. Conclusion

Barley can be further promoted as a human food to partially replace the imported wheat grain. This could allow consumers to benefit from the nutritional value as well as the health claims attributed to barley. In this study, we characterized five barley varieties released in Morocco and two hulless breeding lines for their protein, β-glucan, Fe, Zn, and Se contents. We could identify genotypes suitable for human consumption and recommend the hulless variety Chifaa as a β-glucan biofortified variety for cultivation in Morocco and as a parental germplasm for breeding germplasm combining high β-glucan with high micronutrient contents using Marchouch as selection site for early generations, followed by a multi-environment evaluation of the fixed lines.

## Data availability statement

The original contributions presented in the study are included in the article/supplementary material, further inquiries can be directed to the corresponding author.

## Author contributions

AA, MI, and FE: conceptualization. FE, AJ, and ZK: methodology. FE and ZK: software. AA and MI: validation. FE, ZK, and AA: statistical analysis. FE, AJ, and AA: fieldwork. FE and AE-B: laboratory analysis. FE and AA: writing draft manuscript. AA, MI, and ZK: editing and finalizing. All authors have read and agreed to the submitted version of the manuscript.
